# Characterization of Tungsten Inert Gas (TIG) Welding Fume Generated by Apprentice Welders

**DOI:** 10.1093/annhyg/mev074

**Published:** 2015-10-12

**Authors:** Halshka Graczyk, Nastassja Lewinski, Jiayuan Zhao, Nicolas Concha-Lozano, Michael Riediker

**Affiliations:** 1.Institute for Work and Health, University of Lausanne and Geneva, 1066 Epalinges-Lausanne, Switzerland;; 2.Department of Chemical and Life Science Engineering, Virginia Commonwealth University, Richmond, VA 23284, USA;; 3.SAFENANO, IOM Singapore, Singapore 048622

**Keywords:** gas metal arc welding (GTAW), nanoparticles, occupational exposure, PM4, tungsten inert gas (TIG), welding fumes, workplace air

## Abstract

Tungsten inert gas welding (TIG) represents one of the most widely used metal joining processes in industry. Its propensity to generate a greater portion of welding fume particles at the nanoscale poses a potential occupational health hazard for workers. However, current literature lacks comprehensive characterization of TIG welding fume particles. Even less is known about welding fumes generated by welding apprentices with little experience in welding. We characterized TIG welding fume generated by apprentice welders (*N* = 20) in a ventilated exposure cabin. Exposure assessment was conducted for each apprentice welder at the breathing zone (BZ) inside of the welding helmet and at a near-field (NF) location, 60cm away from the welding task. We characterized particulate matter (PM_4_), particle number concentration and particle size, particle morphology, chemical composition, reactive oxygen species (ROS) production potential, and gaseous components. The mean particle number concentration at the BZ was 1.69E+06 particles cm^−3^, with a mean geometric mean diameter of 45nm. On average across all subjects, 92% of the particle counts at the BZ were below 100nm. We observed elevated concentrations of tungsten, which was most likely due to electrode consumption. Mean ROS production potential of TIG welding fumes at the BZ exceeded average concentrations previously found in traffic-polluted air. Furthermore, ROS production potential was significantly higher for apprentices that burned their metal during their welding task. We recommend that future exposure assessments take into consideration welding performance as a potential exposure modifier for apprentice welders or welders with minimal training.

## INTRODUCTION

Worldwide, ~2 million employees work full-time in welding processes, in addition to numerous other workers who perform welding tasks as part of their occupation (Cena *et al.*, 2014). Workers that participate in welding tasks are exposed to a complex and heterogeneous mixture of welding fumes that consist of metals, metal oxides, gases, and vapours (Lehnert *et al.*, 2012). Metal oxide nanoparticles (NPs) in welding fumes have gained increased attention due to their potential for triggering oxidative stress reactions and contribution to adverse respiratory and cardiovascular outcomes (Antonini *et al.*, 2005). The large surface area of welding fume NPs, matched with a high concentration of metals and organics, favours the formation of reactive oxygen species (ROS) (Wilson *et al.*, 2002). In the alveoli, ROS can react quickly with surrounding tissue, damage cell components, and launch a cascade of local and systemic responses (Riediker, 2007). Such oxidative stress response is an important contributor to acute and chronic vascular and pulmonary diseases (Newby *et al.*, 2014).

When compared to various welding processes, tungsten inert gas (TIG), also known as gas tungsten arc welding (GTAW), has been reported to generate a majority of particles at the nanoscale (Brand *et al.*, 2013). As such, TIG welding fume NPs are of particular occupational research interest due to their potential toxicological properties related to their small size. TIG welding uses a non-consumable tungsten electrode to produce the weld and applies an inert gas (argon or helium) to protect the melting metal from interacting with the atmosphere. A constant-current welding power supply produces energy that is conducted across the arc through a plasma consisting of highly ionized gas and metal vapours. The process grants the operator greater control over the weld than competing processes, allowing for exceptionally clean, strong, and high quality welds (Kou, 2003). As such, TIG welding has become one of the most popular welding methods in various industrial sectors (Fattahi *et al.*, 2013). Despite the increase in TIG welding use and its propensity to generate NPs, there is limited data available for TIG welding fume characterization.

While the International Agency for Research on Cancer (IARC) categorizes welding fumes in Group 2B (possibly carcinogenic to humans), it has also noted that an as-yet unexplained reason for excess lung cancer risks still exists, which demands increased research focused on thorough characterization of specific welding fume components (IARC, 1990; Husgafvel-Pursiainen and Siemiatycki, 2009). However, epidemiological welding studies have often lacked comprehensive exposure assessment, thereby weakening potential causal relationships between exposures to various welding fume components and adverse health effects (Lewinski *et al.*, 2013). Studies performed in industrial settings are often unable to control for numerous welding parameters, making it difficult to disambiguate between heterogeneous aerosol generation. On the other hand, controlled, laboratory based studies often involve robotic welders and acknowledge that welding fume characteristics may substantially differ from occupational environments where fumes are generated by human welders (Cena *et al.*, 2014). The aim of this study was to characterize TIG welding fumes generated by volunteers, while simultaneously ensuring that welding was done in a situation free from workplace contamination or concomitant exposures. We hypothesized that TIG welding fumes would contain metal and metal oxide particles at the nanoscale, and that the TIG welding fume would influence the production potential of ROS. To address these questions, we characterized TIG welding fumes generated by 20 apprentice welders at the breathing zone (BZ; inside of the welding helmet) and at a near-field (NF) station located ~60cm away and at the same height of the welding task. Separate exposure assessments were conducted for each apprentice and included particle concentration, particle size, particle morphology, gravimetric mass, elemental composition, and ROS production potential.

## METHODS

### Welding process

Apprentice welders from Western Switzerland who met inclusion criteria (male, non-smokers, between 16 and 25, no respiratory or cardiovascular disease) were recruited for the study. The study was approved by the Ethics Committee of Canton de Vaud, Switzerland, and was conducted in accordance with the Helsinki Declaration. Volunteers were asked to conduct a 1-h TIG welding task with aluminium OK tigrods (ESAB, 4043, diameter 2.4mm) on 12cm × 12cm × 12cm aluminium cubes (Euralliage, 5005). This welding task represents a standardized exercise performed in welding schools across Western Switzerland. The aluminium tigrods were composed of >92% of Al; 4.5–5.5% Si; 0.6% Fe; 0.05% Mn; 0.05% Mg; 0.1% Cu; 0.15% Ti; 0.1% Zn (ESAB, France) and the aluminium cube metal was composed of >97% of Al; 0.7% Fe; 0.5–1.1% Mg; 0.3% Si; 0.25% Zn; 0.2% Mn; 0.2% Cu; 0.1% Cr (Euralliage, France). TIG welding was done using an ESAB CaddyTig 2200i AC/DC machine (Stucki Soudure SA, Switzerland) supplied with a 98% W, 2% Ce electrode, and 100% argon shield gas. Volunteers wore a non-ventilated welding helmet with auto-darkening lens. The welding task was conducted in a 10 m^3^ exposure cabin with a controlled pulsing ventilation system, an exchange rate of 9.3h^–1^and a high efficiency particulate absorption (HEPA) filter for the incoming and outgoing air (Guillemin, 1975).

### Experimental setup, sampling, and characterization of samples

**Figure 1:**
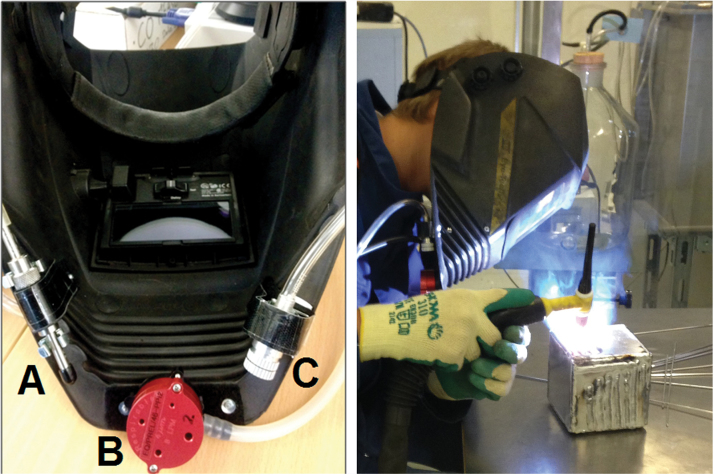
Customized welding helmet with (A) MiniParticle Sampler, (B) parallel particle impactor, and (C) DiscMini inlet impactor shown from inside view; and volunteer wearing customized welding helmet during welding task.

TIG welding fume was characterized at two different locations: one inside of the welding helmet at the BZ of the volunteer, and one at an NF station ~60cm away and at the same height of the welding task. At the BZ, volunteers were equipped with personal sampling monitors that were attached to the inside of the customized welding helmet (Fig. 1).

The customized welding helmet contained a MiniParticle Sampler (INERIS, France) with a copper mesh transmission electron microscopy (TEM) grid attached to an Escort Elf personal pump operating at 0.3 l min^−1^; a PM_4_ parallel particle impactor (PPI, SKC Inc., USA) containing a 37-mm polytetrafluoroethylene (PTFE) filter (Pall Life Sciences, USA), attached to a Leland Legacy personal pump (SKC Inc., USA) with flow rate of 8 l min^−1^; and the inlet impactor (0.8-μm cutoff) of a DiscMini particle counter (Matter Aerosol, Switzerland). Logging interval of the DiscMini was 1 s.

Air flow of all pumps was controlled before and after sampling, and operated for 60min (i.e. the length of the welding period). For *N* = 5 TIG welding experiments, we also collected TEM grids in parallel MiniParticle Samplers, but operating at lower airflow (0.05 l min^−1^) for much shorter collection time (30 s). Exposed TEM grids were imaged using a Phillips CM-20 transmission electron microscope operating at 80 keV (Phillips, Holland).

The mass concentration of the collected PM was determined via gravimetric analysis. After storage in standard atmosphere for at least 24h, the PTFE filters were weighed before and after exposure with a XP2U Ultra Micro Balance (Mettler-Toledo, Switzerland). Static charges were discharged using a 210Po ionization source. Post-exposure mass was measured, and the mass of the collected PM was calculated by taking the difference between the pre- and post-exposure mass values and correcting with blank filters to adjust for temperature-related variations. After gravimetric mass analysis, filters were stored in dedicated PetriSlide dishes (Merck Millipore, Germany). Each filter was analysed by X-ray fluorescence (XRF) spectroscopy following the EPA Compendium Method IO-3.3 (Cooper Environmental Services, USA). A total of 48 non-gaseous elements were analysed by XRF analysis. Elemental composition of the welded cubes were assessed by energy dispersive spectroscopy (EDS) using a FEI Quanta 250 scanning electron microscope (FEI, USA) and a Bruker Nano X-ray microanalysis system (Bruker Nano Analytics, Germany) operating at 20 keV.

The NF characterization station included a second PM_4_ PPI and a 10 l buffer bottle to which a scanning mobility particle sizer (SMPS: Grimm Ainring, Germany, models 55-40-25 DMA and 5.403 CPC) and a second DiscMini particle counter were connected. The SMPS measures particles in a size range from 10 to 1110nm in 31 different size channels and in concentrations up to 10^7^ particles cm^−3^, and has been widely used as the standard for measuring airborne particle size distributions due to its ability to conduct differential mobility analysis of various particles (Wang and Flagan, 1990; Coquelin *et al.*, 2013; further information about SMPS sizing methods is included in Supplementary Material, note S1). The SMPS was set to fast scan mode with measurement cycles of 3.5min. The DiscMini had the same specifications as at the BZ. To confirm the absence of larger particles and for quality control purposes, we also measured the particle size distribution with an optical particle counter (OPC, Grimm, Ainring, Germany, model 1.109), which sizes particle diameters within the size range 0.25–32 µm in 31 size channels, on *N* = 5 random welding days. The buffer bottle used at the NF station served to reduce short-term exposure fluctuations, which is necessary for the SMPS and OPC size characterization.

The PTFE filters were treated as described at the BZ. The NF characterization station also included an O_3_ gas monitor with an LOQ of 0.001 p.p.m. (Aeroqual, New Zealand), a NO_2_ gas monitor with a LOQ of 0.1 p.p.m. (Dragerwerk, Germany), CO_2_ gas monitor (Dragerwerk, Germany), CO gas monitor with a LOQ of 0.1 p.p.m. (Dragerwerk, Germany) and a NO gas monitor with a LOQ of 0.1 p.p.m. (Dragerwerk, Germany). Concentrations of these gases were not expected to be elevated due to the low current intensity of TIG welding (DGUV, 2009), but were nonetheless measured on 30% of the study days for confirmatory purposes.

### Reactive oxygen species (ROS)

The acellular ROS detection method applied was adapted from a previously published protocol (Zhao and Riediker, 2014) using fluorescent dye 2′7-dichlorodihydrofluorescein (DCFH). Briefly, the 37-mm PTFE filters with welding samples were carefully rolled up and placed into individual polypropylene conical tubes filled with DCFH-horse radish peroxidase (HRP) working solution. The tubes were protected from light and sonicated for 15min at constant 37°C. Sonication blank control was obtained by sonicating pure DCFH-HRP solution. A 96-well multiple plate reader (Infinite M200, TECAN; DCF excitation/emission at 485/530nm), kept at constant 37°C, was set to a dynamic fluorescence reading pattern, performing one reading per minute for 60min. The equivalence of H_2_O_2_ was converted from calibration curves. The final values were calculated by subtracting sonication blank from raw sample data.

### Data treatment and statistical analysis

Data of all real-time measurements were processed with the standard software delivered with the corresponding device and imported into STATA 13, which was used for statistical analysis (StataCorp LP, College Station, TX, USA). SMPS manufacturer software (Aerosol Instrument Manager®, Version 1.34, GRIMM aerosol technik) was used to obtain the total number concentration and geometric mean diameter (GMD) of the aerosol for the welding periods. The statistical analysis was computed for the entire particle size distribution and was not limited to a specific size range. The DiscMini measurements of the welding periods were averaged to obtain total number concentration and GMD. Exploratory analysis was conducted on reported variables in total and for each volunteer. Particle numbers and ROS production potential values were log10-transformed to conduct linear regression analysis. Differences of particle number concentration (PNC) and GMD between particle counting instruments were assessed with the Kruskal–Wallis rank test. Direct comparisons of additional particle characterization data were assessed with either matched or unmatched non-parametric tests. We used a *P*-value of <0.05 to define statistical significance. Holm corrections for multiple comparisons were applied.

## RESULTS

A total of 23 volunteers were recruited from local welding apprentice schools, of which 3 were excluded for medical reasons. This resulted in 20 volunteers participating in the 1-h TIG welding task, all of which were healthy, non-smoking male welding apprentices between the ages of 16 and 25 from Western Switzerland.

### Particle size distribution

Real-time data of particle size distribution were analysed separately for each volunteer to ensure consistency of measurements as well as cumulatively to present summary statistics by each instrument. Summary statistics for geometric means and geometric standard deviations (GSD), range, and coefficient of variation (CV) are provided for PNC and particle diameter for each instrument in Table 1.

**Table 1.** Summary of geometric mean (GM) and geometric standard deviation (GSD), range and coefficient of variation (CV) of particle number concentration (PNC) and geometric mean diameter (GMD) of SMPS and DiscMini at the near-field (NF) and breathing zone (BZ). Data from the DiscMini at both BZ and NF station were available for all 20 volunteers, while SMPS data was available for 19 volunteers due to instrument malfunction for one volunteer.

	SMPS (NF)	DiscMini 1 (NF)	DiscMini 2 (BZ)
*Number of valid observations*	19	20	20
**PNC (particles cm^−3^)**			
GM of PNC	7.74E+05	1.10E+06	1.69E+06
GSD	1.6	1.86	2.42
PNC Min	2.70E+05	5.23E+05	8.69E+05
PNC Max	1.28E+06	2.89E+06	3.85E+06
CV	60%	86%	142%
**Size (nm)**			
GMD	69	51	45
GSD	1.89	1.48	1.61
GMD Min	32	32	29
GMD Max	170	112	108
CV	89%	48%	61%

The GMDs measured between all three particle counters were not statistically different from each other. The particle size distribution at the BZ across all volunteers showed that a majority (92%) of the particles had measured GMDs below 100nm, and 50% of the particles had measured GMDs below 41nm. We compared results from the two DiscMinis at separate locations and found that the PNCs and GMDs from DiscMini 1 (NF) and DiscMini 2 (BZ) had a positive correlation of *r* = 0.80 and *r* = 0.84, respectively.

The particle size distribution as measured by the SMPS suggests that a majority of the particles were below 100nm, with very few particles in the 1000nm range (Supplementary Fig. S1 and Table S1). These results were supported by the OPC data that showed very few particles above 1000nm (Supplementary Fig. S2).

### Morphology

**Figure 2:**
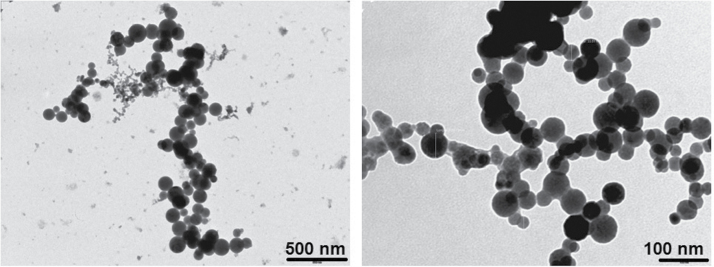
TEM images of collected TEM welding NPs during 60-min collection time at 0.3 l min^−1^.

Analysis of TEM images from grids collected for 60min at 0.3 l min^−1^ shows primary welding fume particles in the nanoscale that were spherical and often formed chainlike agglomerates with fractal geometry (Fig. 2). TEM images analysed from grids collected for 30 s at 0.05 l min^−1^ show smaller agglomerates in size ranges that correspond with SMPS and OPC particle count data (Supplementary Fig. S3a and S3b).

### Gravimetric mass

Analysis of PTFE filters from the BZ were based on 20 filters; one from each volunteer. Analysis of PTFE filters from the NF was available for 18 volunteers. The median gravimetric mass at the BZ was 0.716mg m^−3^ (25th–75th percentiles: [0.351–1.271]) and 0.672mg m^−3^ at the NF (25th–75th percentiles: [0.403–1.77]). There was no statistically significant difference between measured mass values at the BZ and at the NF (Supplementary Fig. S4).

###  Chemical composition of TIG welding fume

**Figure 3:**
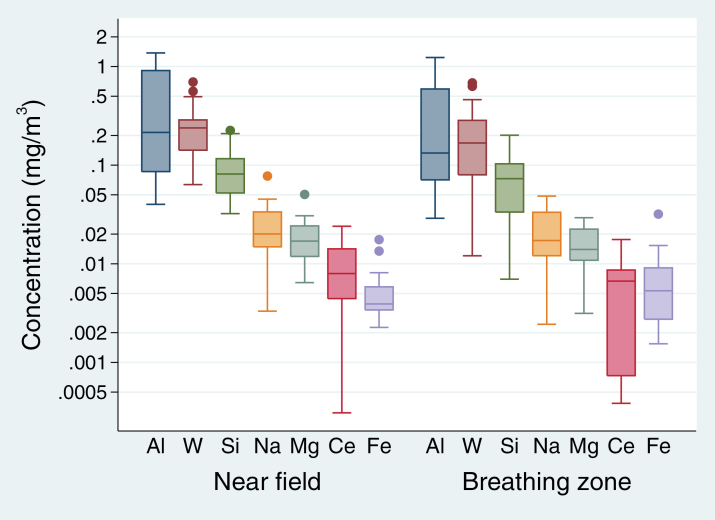
Boxplot of the elemental composition of particles at the NF and BZ, showing the interquartile range (IQR) as the length of the box, the median (line dividing the box), the whiskers spanning all data points within 1.5 IQR of the nearer quartile, and outliers (filled-in circles). Results are presented in mg per unit air. There was no statistically significant difference in elemental concentrations between the two sampling locations.

The most abundant elements (w/w concentration >1%) present in all assessed filters were, in descending order: Al (43% w/w), W (35.1% w/w), Si (12.4% w/w), Na (2.8% w/w), Mg (2.3% w/w), Ce (1.3% w/w), and Fe (1.2% w/w). Median concentrations at the BZ were 0.231mg m^−3^ Al, 0.130mg m^−3^ W, 0.067mg m^−3^ Si, 0.017mg m^−3^ Na, 0.014mg m^−3^ Mg, 0.007mg m^−3^ Ce, and 0.005mg m^−3^ Fe (Fig. 3). There was no statistically significant difference between the median concentrations of the elements at the two sampling locations.

Mean concentrations of O_3_, CO_2_, and NO_2_ were above the detection limit; however, NO and CO were not detected. The average concentration of O_3,_ CO_2_, and NO_2_ was 0.009±0.008 p.p.m., 650±48 p.p.m. and 0.3±0.2 p.p.m., respectively. See Supplementary Table S2 for full gas concentration summary.

### ROS analysis

Mean ROS production potential at the BZ was 16.89±15.39 nmol m^−3^ and 13.68±17.28 nmol m^−3^ at the NF. There was no statistically significant difference between the ROS production potential concentrations collected at the two sampling locations (see Supplementary Table S3 for full ROS production potential results). We found that ROS production potential was correlated with several metals; however, after applying Holm correction for multiple comparisons, all of these correlations were no longer significant. Full results of the uncorrected and corrected *P*-values are included in Supplementary Table S4.

### Welding task performance and cube oxidation analysis

**Figure 4:**
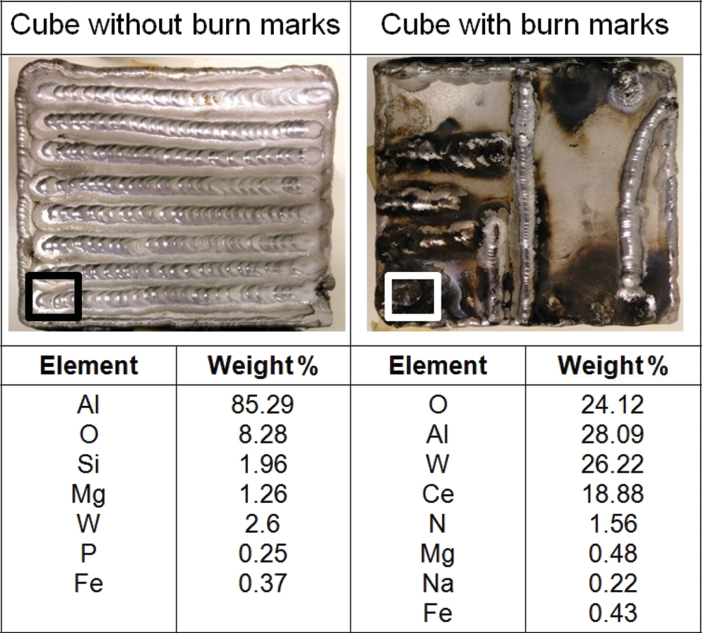
Comparison of two completed TIG welding tasks of two different volunteers, showing differences in TIG welding performance in regards to the quality of the weld. The square on the bottom left corner of each cube indicates the area where EDS analysis of the surface composition was performed. Elemental concentrations are expressed as percentages of overall weight.

Clear interpersonal variability was observed for TIG welding performance in regards to the quality of the weld when assessing the welding line formation and oxidation burn marks on the aluminium cube (Fig. 4). Evidence of these burn marks allowed separating the participants into two distinct groups, one without any evident burns on their cube (*N* = 12) and one with significant burn marks (*N* = 8). A sample of the two cubes shown in Fig. 4 was assessed with EDS analysis to better understand potential differences in elemental composition. The most abundant element of the cube without burn marks was Al, while the top most abundant elements of the cube with evidence of burns were Al, O, W, and Ce.

**Figure 5:**
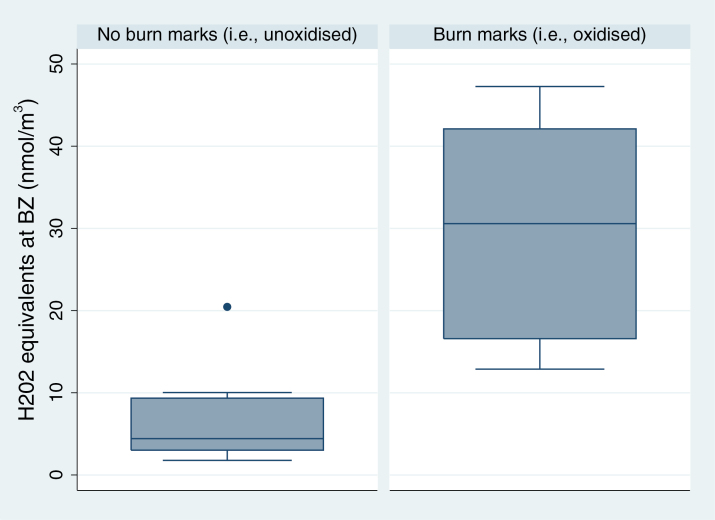
Boxplot showing IQR (box), median (line dividing box), whiskers spanning all data points within 1.5 IQR of the nearer quartile, and outlier (filled-in circles) of reactive oxygen species (ROS) production potential values (shown as H_2_O_2_ equivalents per filter in nmol) at the breathing zone (BZ) of two groups of volunteers: those that completed their task without any evidence of burn marks (metal not oxidized) and those that completed their task with evidence of burn marks (metal oxidized).

ROS production potentials were also compared between volunteers with and without burn marks (i.e. oxidization) on their cube. We found that mean ROS production potential of volunteers with visible burn marks on their cube were significantly higher than the mean ROS production potential of the volunteers without burn marks on their cube (Fig. 5). We did not find a correlation between ROS production potential and metal concentration within these two groups of volunteers. There was no significant difference between the two groups of volunteers in regards to particle size, particle number concentration, and gravimetric mass. There was also no significant difference between the two groups for metal concentrations, except for aluminium, which was significantly higher for the group with burn marks on their cube.

## DISCUSSION

### Particle size distribution and morphology

Characterization of TIG welding fume during the 1-h welding period suggests that apprentice welders are exposed to high number concentrations of aerosolized particles with GMDs almost exclusively below 100nm at the BZ when practicing for their trade. We observed strongly elevated PNCs despite a well-ventilated working environment free of other particle sources. The few studies that have characterized the particle size of aluminium TIG welding fume report mean diameters in the sub-micron range (Dasch and D’Arcy, 2008; Berlinger *et al.*, 2011; Brand *et al.*, 2013; Pohlmann *et al.*, 2013; Debia *et al.*, 2014). Berlinger *et al.* (2011) measured aluminium TIG welding fume using a SMPS and reported mobility sizes ranging between 15 and 160nm, with a majority of particles below 100nm. Pohlmann *et al.* (2013), using a condensation particle counter to measure aluminium TIG welding fume reported a number median of mobility size distribution of 63nm, closely matching our GMD of 69nm as measured by SMPS. Our results are also in agreement with the particle size distribution findings of Lehnert *et al.*, 2012 and Brand *et al.*, 2013, which involved measuring aluminium TIG welding fumes isolated from other processes. Specifically, Brand *et al.* (2013), characterized TIG welding fume particles with a fast mobility particle sizer (FMPS) that uses the same electrical mobility measuring method as that of the SMPS used in our study. The study found that TIG welding fume particles, measured at 60cm away from the welding source, are almost exclusively <100nm (99%), with at least 90% <50nm. However, the size distribution mode of 214nm measured in an apprentice welding school and 0.88 μm measured in an industrial setting differed from our particle size measurements (Dasch and D’Arcy, 2008; Debia *et al.*, 2014). We observed in pilot measurements at the welding schools of our volunteers that the influence of other welding processes while characterizing aluminium TIG welding fumes resulted in greater particle size variability and larger particle size than in a controlled setting. As such, the differences observed in these two field characterization studies may have been in part related to the presence of particles from other welding or metal working processes in the school and the industrial setting, as well as the use of impactor-based devices versus condensation particle counters.

In regards to particle morphology, our TEM observations of TIG welding fume particles are in agreement with previous studies, specifically with the finding of agglomerated chains of primary nanosize particle (Jenkins *et al.*, 2005). We found that the TEM images from grids collected at a low sampling airflow (0.05 l min^−1^) and at a lower sampling time (30 s) were in agreement with our continuous sampling measurements as reported by the DM, SMPS, and OPC. These results suggest that there are very few micron size agglomerates present in the air. Evidence from the literature supports these findings. In a comparison of the agglomeration rate of various types of welding, Pohlmann *et al.* (2013) found that the agglomerate emission rate for TIG welding was much lower than in other welding processes. Brand *et al.* (2013) further reported that the agglomeration of primary particles in TIG welding is much slower than in other welding processes due to different coagulation dynamics after primary particle formation related to the low mass emission rates of TIG welding (0.01–0.05mg s^−1^). The authors further reported that at 60cm away from the welding source, TIG welding fume particles are still in the 10nm size range, and this fraction of nanoscaled particles is stable for at least some minutes. Our results show that a majority of TIG welding particles are below 100nm; however, we do not exclude the possibility that larger particles, those agglomerated chains that are between 100 and 1000nm, may have been captured on the TEM grid as shown in Fig. 2, and may be contributing to our measured mass.

### Measurement locations

We found that the mean PNC at the BZ was 54% higher than the mean PNC at the NF, and median gravimetric mass at the BZ was 7% higher than at the NF. Moreover, mean ROS production potential concentrations were 23% higher at the BZ than at the NF. There are only a few published reports that compare differences in welding fume exposure from the BZ and NF and these reports present conflicting results. Goller and Paik (1985) reported that fume mass concentrations from flux cored arc welding (FCAW) inside of the welding helmet were 36 to 71% of those measured outside the helmet. Similarly Cole *et al.* (2007) found that fume mass concentration reduction ranged from insignificant to over 99% between outside and inside the welding helmet of a mannequin placed near robotic gas metal arc welding (GMAW) and GTAW. Liu *et al.* (1995) and Harris *et al.* (2005) showed that there was generally little difference between SMAW fume mass concentrations inside and outside the helmet, with significant reductions only at relatively high mass concentrations (>20mg m^−3^). The lack of reduction and high variability in reduction reported under different exposure settings confirm that a non-ventilated welding helmet is not sufficient to provide for respiratory protection. Our gravimetric results further confirm the conclusion of Harris *et al.* (2005) that for welding situations of low to moderate mass concentration exposures, there may be little difference between concentrations inside and outside of the welding helmet.

### Chemical composition

The most abundant elements measured at both the BZ and at the NF were Al, W, Si, Na, Mg, and Ce, which represent a mixture of elements from the welding piece, the consumable filler rod and the electrode. Aluminium being one of the most abundant elements measured was expected and easily justified by the combustion of the aluminium filler rod. However, high concentrations of tungsten in TIG welding fumes have not been previously reported. One study that measured 49 metals including tungsten by ICP-MS from mild steel TIG welding fume found no tungsten (Chang *et al.,* 2013).

Elevated tungsten concentrations for certain volunteers most likely resulted from combustion of the tungsten electrode during the welding task. This was affirmed by the presence of cerium, the tungsten-electrode alloy metal, as one of the top most abundant elements present, as well as evidence of tungsten and cerium on oxidized cubes. When TIG welding correctly, the electrode does not combust and is considered to be non-consumable. However, electrode consumption has been reported to occur during operation of reverse polarity, accidental contact of the tungsten electrode to the welding puddle, or incomplete shielding gas coverage (Jeyaprakash *et al.*, 2015). Apprentice welders are specifically instructed to avoid contact of the electrode to the welding puddle, and to raise the torch so that it is 0.4–0.6cm away from the work piece. As polarity and shielding gas was correctly established for all welders in our study, our findings suggest that composition of the welding fume particles was influenced in part by the ability of the welder to avoid contacting the electrode to the welding puddle.

Chronic human exposure to tungsten is reported to primarily involve respiratory effects such as pulmonary fibrosis, yet the carcinogenic potential of tungsten has not been adequately characterized (ATSDR, 2005). Limited inhalation toxicology data are available on tungsten with one sub-chronic rodent study reporting that an inhaled dose over 300 times the threshold limit value for tungsten (5mg l^−1^) resulted in increased blood tungsten concentrations but no apparent toxicity (Rajendran *et al.*, 2012; Witten *et al.*, 2012; Lemus and Venezia, 2015). Literature on tungsten metal, tungsten carbide, and tungsten disulfide NPs have reported tungsten as relatively chemically inert when measuring cell viability, but not when measuring oxidative stress (Lemus and Venezia, 2015). Our findings of elevated tungsten exposures highlight the need for further research on the potential health effects from occupational tungsten exposures in TIG welding. As it has been recommended that increased research on age-related differences in susceptibility and biokinetics of tungsten exposure is conducted (ATSDR, 2005), research on apprentice TIG welders may be particularly important.

To date, research on tungsten exposure via welding has focused specifically on thorium exposure from thoriated tungsten electrodes due to thorium’s radioactivity (Jankovic *et al.*, 1999; Gäfvert *et al.*, 2003). Tungsten electrodes alloyed with 1–4% thorium oxide are often used in lieu of ceriated or lanthanated electrodes as thoriated electrodes facilitate arc starting, cause less weld metal contamination and improve arc stability (Gäfvert *et al.*, 2003). Despite ventilated and controlled working environment, we measured cerium, the tungsten-electrode alloy metal, in both BZ and NF filters, as well as on the oxidized cube. While we cannot use our results to assume or predict that apprentice welders using thoriated electrodes would be exposed to similar levels of the electrode alloy metal, it is reasonable to recommend that apprentice welders, or welders with little practice, do not use radioactive thoriated electrodes due to increased risk of electrode consumption before properly mastering their technique. This recommendation is especially prudent given the widespread availability of non-thoriated tungsten electrodes for use as viable alternatives.

It is well documented that TIG welding generates much lower ozone concentrations than other welding types (DGUV, 2009). Ozone concentrations in our study were at ambient air levels and below what has been previously reported in TIG welding (Burr *et al.*, 2005; Liu *et al.*, 2007; DGUV, 2009; Azari *et al.*, 2011). As such, the contribution of TIG welding to O_3_ exposure in our study was very small.

### ROS production potential

We investigated the ROS production potential of welding fume particles using the DCFH assay, which has been widely applied for non-specific ROS detection (Venkatachari *et al.*, 2007; Jiang *et al.*, 2008; Aranda *et al.*, 2013; Zhao *et al.*, 2014) and found to be relatively inexpensive and robust. Regarding the reported limitations of this assay in the literature (Hoet *et al.*, 2013; Pal *et al.*, 2014), we carefully applied the strategies published in the study by Zhao and Riediker (2014) to achieve better performance of the assay and avoid artefacts, such as to always keep an appropriate control to exclude ROS produced by self-oxidation of the reagents and analyse the standards with samples at the same time point. We would also like to emphasize that the ROS potential reported here were from the functional groups that respond to DCFH-HRP reactants in an acellular condition. We do not exclude the possibility that ROS species that are not responsive to DCFH can be generated with welding fume and pose oxidative stress, which was not detected with the DCFH assay.

The wide application of the DCFH assay facilitated the direct comparison between the present study and available literature. A comparison of our results to ROS concentrations measured in previous ambient aerosol studies in units of equivalent nmol H_2_O_2_ per cubic meter air is presented in Table 2 (Hung and Wang, 2001; Venkatachari *et al.*, 2005a,b, 2008; See *et al.*, 2007; Wang *et al.*, 2011; King and Weber, 2013; Khurshid *et al.*, 2014). Put into context with available literature, our results show that the ROS production potential exposure resulting from the TIG welding fumes in our study may exceed that of ambient air PM from various geographical regions, as well as in traffic-polluted air PM (See *et al.*, 2007).

**Table 2.** Average ROS production potential measured in previous aerosol studies, presented in nmol of H_2_O_2_ per cubic meter air, and in inhaled nmol per hour.

ROS prod. potential(nmol H_2_O_2_ m^−3^-air)	Inhaled ROS(nmol h^−1^)
		Source location and type
*TIG welding fume at BZ*	*16.89*	*6.08*
*TIG welding fume at NF*	*13.68*	*4.92*
Taipei (Taiwan) sidewalk	0.54	0.19
Singapore (Singapore) ambient	5.71	2.06
Singapore (Singapore) traffic	15.1	5.44
Rubidoux, CA (USA) ambient	5.89	2.12
Flushing, NY (USA) ambient	0.87	0.31
Rochester, NY (USA) ambient	8.3	2.99
Atlanta, GA (USA) urban ambient	0.26	0.09
Atlanta, GA (USA) rural ambient	0.14	0.05
Austin, TX (USA) outdoor	1.41	0.51
Austin, TX (USA) inside homes	1.37	0.49

Italicized values denote the ROS production potentials measured in this study.

Given that the normal human breathing rate is ~12 breaths per minute, with about half a litre of air inhaled with every breath (Wilkerson, 2001), we calculated the approximate dose of ROS production potential inhaled during 1h depending on the exposure scenario (Table 2, second column). Moreover, it has been reported that smoking one commercial cigarette (Marlboro gold) generates an average of 90.3 nmol of ROS, and inhalation of sidestream smoke results in exposure to 19.7 nmol of ROS (Zhao and Hopke, 2011). By analogy, exposure to ROS during 3h of TIG welding could be equivalent to sidestream exposure from one commercial cigarette, while 15h of TIG welding (~2 full working days) could be equivalent to its mainstream smoke.

Elevated ROS production potential concentrations may be explained by the high energy of the welding arc that results in the generation of unstable and highly reactive metal oxide particles capable of forming OH radicals, precursors, and initiators of many forms of ROS (Leonard *et al.*, 2010). Antonini *et al.* (1998) found that freshly generated welding fumes are more reactive than aged welding fume due to higher ROS concentrations on the fume particle surface. Chang *et al.* (2013) further demonstrated that ROS activity of welding fume particles was size dependent, with particles of the fine and ultrafine range (PM0.1–2.5 and PM0.1) having higher ROS concentrations when compared with course PM10 (PM2.5–10). The authors found that ROS concentrations for each particle range were not always in line with the mass concentration levels from gravimetric analysis, giving a clear indication that a mass dose metric might not be a good metric for toxicity related to welding fumes.

ROS production potential was significantly higher for apprentices that burned their metal cube in the welding task. When TIG welding with poor technique, the electrode touches the molten weld pool and surface tension pulls the aluminium up onto the hot tungsten electrode. The extreme heat of the electrode causes the metal to vaporize and form a large, widely scattered black oxide layer (Jeffus, 2004). Results of EDS analysis showed higher oxygen content on the surface of the burned cube which supports the presence of the black oxide layer described by Jeffus (2004), while increased tungsten and cerium content on the surface of the burned cube confirms contact of the electrode to the cube. In addition, higher concentrations of aluminium in the air for the apprentices that burned their cube supports the increased vaporization of aluminium from the hot electrode. Leonard *et al.* (2010) previously found that the elemental composition of the welding fume may have an impact on the ROS production potential. Moreover, several transition metals, such as Fe, Cr, Co, Mo, Mn, and Cu, have been shown to play important roles in ROS generation and redox chemistry (Mills *et al.*, 2009; Valko *et al.*, 2005). Specifically, Pal *et al.* (2014) found an association between ROS production potential values and Fe, Cr, Co, Mo, Mn, and Ni using the DCFH assay. While we did find a correlation between ROS production potential and several transition metals (e.g. Fe, Zn, Y, Ag), all significance disappeared when applying the Holm correction for multiple comparisons. It is worth noting that our results do not imply that these metals are not important for ROS formation, but rather, in our samples, they did not seem to play a major role, which could be explained by their low mass contribution. Of the metals that Pal *et al.* (2014) found a correlation between ROS production potential values, the most abundant one in our study was Fe. However, the w/w concentration of Fe was only 1.2% with a very low median concentration of 0.005mg m^−3^. Therefore it is possible that our findings diverge from Pal *et al.* (2014) results due to our low concentrations of the transition metals relative to other more abundant elements. The correlation results presented may therefore be underestimated, and the effect of certain transition metals may have a greater effect on ROS production potential than found here.

Overall it is unclear whether ROS production potentials were influenced by one particular metal. Rather, the correlation between cube oxidation and increased ROS production potentials may be in part explained by increased generation of unstable metal oxides that are capable of temporarily attaining different and more reactive transition or valence states (Leonard *et al.*, 2010). The question of the role of the oxidation state of welding particles, whether highly oxidized functional groups or the valence of the involved ions, as drivers of ROS production potential warrants further investigation in future studies. Nevertheless, it is important to emphasize that welding performance in regards to the quality of the weld was related to the ROS generation potential of the fume, thereby signifying a potential determinant for exposure. As induction of ROS by NPs is a key event in toxicological cellular response and an important contributor to acute and chronic vascular and pulmonary diseases, it is imperative also with regard to health protection, and not only regarding quality of the weld, that proper welding techniques are used in order to avoid excess oxidation. This finding can be applied to other welding cohorts, and it is recommended that level of training and welding performance in regards to the quality of the weld is considered as a factor for future exposure assessments. Apprentice welders or welders with minimal training should be cognizant of the importance of proper welding techniques. This is particularly important as TIG welding fume is generally less visible than other welding fume when produced, resulting in assumptions among welding trainers that it is cleaner and less dangerous (personal communication by trainers of the Center for Professional Training, Lausanne, 2014). The elevated concentrations of NPs generated in our study highlight the importance of clarifying this misconception. The induction of ROS in TIG aluminium welding as mechanism for respiratory and neurological effects has yet to be thoroughly investigated. It is recommended that further research is carried on ROS generation in aluminium TIG welding and resulting toxicological effects following inhalation, particularly for apprentice welders and welders with minimal training.

## CONCLUSION

In this study we characterized TIG welding fumes in a controlled environment while maintaining an occupationally relevant human factor for fume generation. We showed that apprentice welders are exposed to high concentrations of welding fume particles that consist almost exclusively of particles with GMDs <100nm. Moreover, despite a ventilated and well controlled setting, 1-h TIG welding tasks generated average ROS production potentials that exceeded averages previously found in traffic-polluted air. Furthermore, ROS production potential was significantly higher for apprentices that burned their metal during their welding task. We recommend that future exposure assessments take into consideration welding performance in regards to quality of the weld as a potential exposure modifier for apprentice welders or welders with minimal training. Increased research needs to be carried out to assess the health effects of apprentice welders exposed to TIG welding fumes.

## SUPPLEMENTARY DATA

Supplementary data can be found at http://annhyg.oxfordjournals.org/.

## FUNDING

This work was supported by the Swiss National Science Foundation, NRP 64 Project, Grant No. 406440_131282 to M.R. for doctoral research funding of H.G. and the Leenaards Foundation, Nested Research Projects Grant No. 3641 for research funding of N.L.

## Supplementary Material

Supplementary Data
