# Supplementary material for: Characterization of Tungsten Inert Gas (TIG) Welding Fume Generated by Apprentice Welders
Source: Ann Occup Hyg. 2015 Oct 12;60(2):205–19. doi: 10.1093/annhyg/mev074 (PMC4738234; doi:10.1093/annhyg/mev074)
Supplement: Supplementary Data [file supp_60_2_205__index.html]

Characterization of Tungsten Inert Gas (TIG) Welding Fume Generated by Apprentice Welders — Characterization of Tungsten Inert Gas (TIG) Welding Fume Generated by Apprentice Welders — Supplementary Data 

# Characterization of Tungsten Inert Gas (TIG) Welding Fume Generated by Apprentice Welders

## Supplementary Data

Data files

- Supplementary Data - Supplementary Data
