# Supplementary material for: Characterization of Tungsten Inert Gas (TIG) Welding Fume Generated by Apprentice Welders
Source: Ann Occup Hyg. 2015 Oct 12;60(2):205–19. doi: 10.1093/annhyg/mev074 (PMC4738234; doi:10.1093/annhyg/mev074)
Supplement: Supplementary Data [file supp_mev074_Graczyk_SupplementalInformation_UPDATED.pdf]

Characterization of Tungsten Inert Gas (TIG) Welding Fume Generated by Apprentice Welders

Halshka Graczyk, Nastassja Lewinski, Jiayuan Zhao, Nicolas Concha-Lozano and Michael Riediker

SUPPLEMENTARY MATERIAL

Note S1 on SMPS sizing methods: The SMPS has been widely used as the standard for measuring airborne particle size distributions due to its ability to conduct differential mobility analysis of various particles (Wang and Flagan, 1990, Coquelin et al., 2013). This method is based on the physical principle that the ability of a particle to traverse an electric field (electrical mobility) is fundamentally related to particle size. The DMA measures the size of entering particles by charging them with a known number of charges and then measuring the distance travelled (= the speed of the particles) when an electric field is applied. Particle size is then calculated from the mobility distribution.

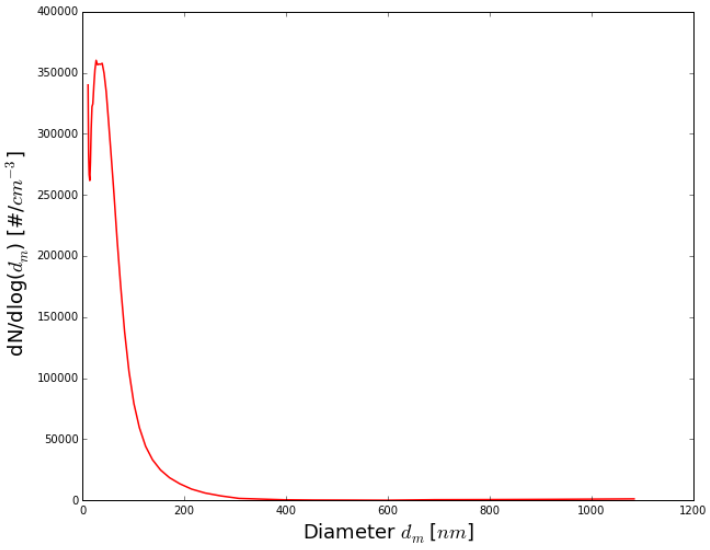

Figure S1. Particle size distribution as measured by SMPS for a representative welding experiment, corresponding with Figure 2 (TEM image), in the manuscript. SMPS results show a peak in particle concentration in the range below 100 nm, with very few particles in the 200-1000nm size range.

Particle concentration across selected SMPS sizing channels

**SMPS Sizing Channel (nm)**	**32.4**	**35.5**	**38.9**	**42.6**	**……**	**692.1**	**801.4**	**930.5**	**1083.3**
**Particle concentration (#/cm^3)**	357142	356938	357703	349623	……	518	623	828	1181

Table S1. Particle concentration across selected SMPS sizing channels corresponding to supplementary figure S1, showing that the concentration of particles in the 1000nm range are orders of magnitude smaller than the concentration of particles in the range below 100nm.

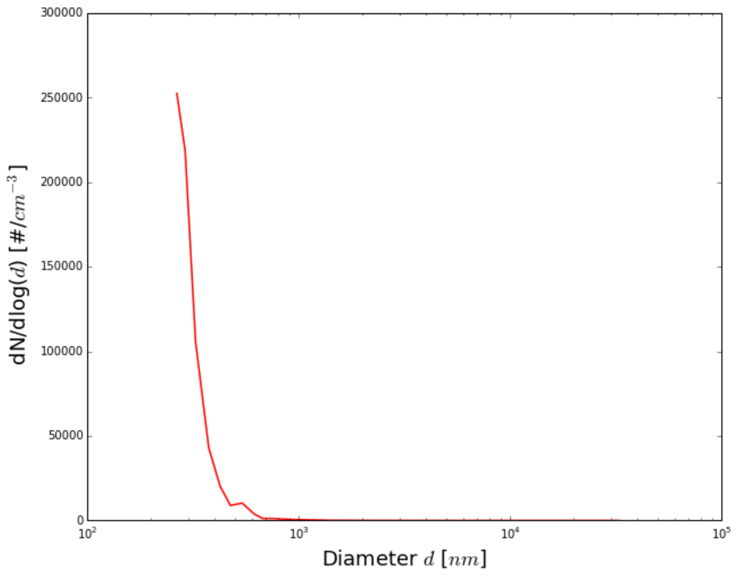

Figure S2. Average particle size distribution of N=5 TIG welding experiments as measured by the OPC, with x-axis (diameter) on log scale. OPC results are agreement with our SMPS data and confirm that a large majority of measured particles were below 1000nm.

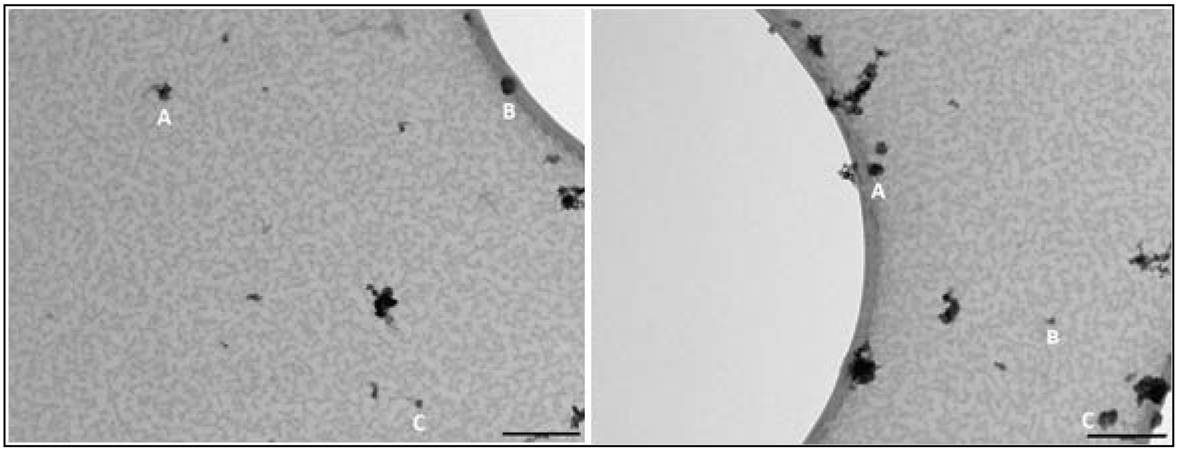

Figure S3a and S3b. TEM image of collected TIG particles at 0.05 L/min for 30 seconds. Letters show examples of mean diameters (Average of 3 replicate measurements) with ImageJ software (NIH, USA). For figure 3a (left): A=40 nm; B=45nm; c=20 nm. For figure 3b (right): A= 43 nm; B=29nm; c=55 nm. Scale bar=200nm.

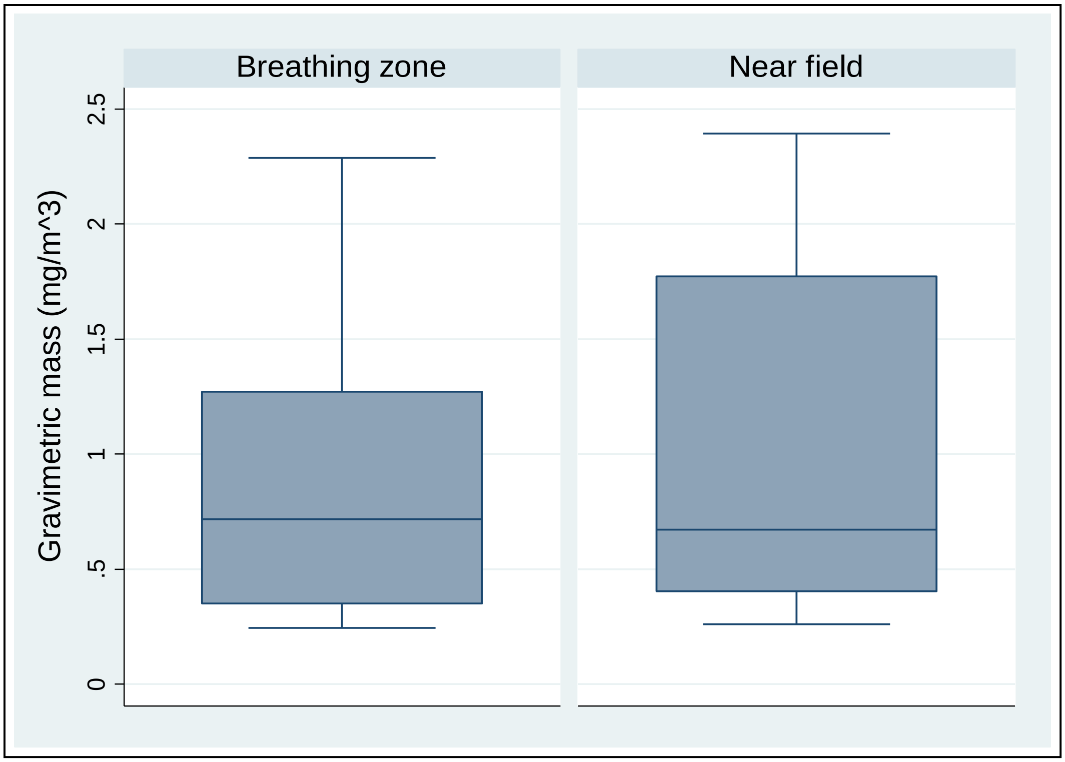

Figure S4. Boxplot of the gravimetric mass at the breathing zone and near field, showing the interquartile range (IQR) as the length of the box, the median (line dividing the box), and whiskers spanning all data points within 1.5 IQR of the nearer quartile. There was no significant difference between the two sampling locations.

**Gas measurements summary (ppm)**						
Gas	**LOQ**	**Mean**	**Min**	**Max**	**Std**	**Background**
**O_3**	0.001	0.009	0	0.165	0.008	0.001
**CO_2**	nc	650	510	816	48	511
**CO**	1	0.0	0.0	0.0	0.0	0
**NO**	1	0.4	0.0	5.0	0.8	0
**NO_2**	0.1	0.3	0.0	1.0	0.2	0.0

Table S2. Concentrations of gases measured during TIG welding period (N=6). The background indicated the mean value 10 minutes before and after the exposure.

**Mean H_2O_2 equivalents (nmol/m^3-air)**					
**Filter**	**Mean**	**Std. Dev.**	**Min**	**Max**	**CV**
**BZ**	16.89	15.39	1.78	47.26	91%
**NF**	13.68	17.28	1.05	62.66	126%

Table S3. Summary of ROS production potential from near field (NF) and breathing zone (BZ), presented in nanomoles (nmol) of H2O2 equivalents per cubic meter air.

Element	Uncorrected p-value	Holm corrected p-value
	**Transition Metals**	
**W**	.253	1
**Fe**	.028	.533
**Au**	.408	1
**Zn**	.019	.386
**Y**	.036	.635
**Ti**	.928	1
**Mn**	.101	1
**Mo**	.165	1
**Cu**	.099	1
**Co**	.294	1
**Zr**	.501	1
**Cr**	.564	1
**Ni**	.820	1
**V**	.172	1
**Ir**	.938	1
**Nb**	.413	1
**Ta**	.566	1
**Ag**	.047	.797
**Sc**	.694	1
**Hf**	.942	1
**Non-transition metal elements contributing to 1% of total mass**		
**Al**	.378	1
**Si**	.205	1
**Na**	.167	1
**Mg**	.181	1
**Ce**	.177	1

Table S4. Uncorrected and Holms-corrected p-values for correlation between ROS production potential and transition metal concentration and non-transition elements contributing to 1% of the total mass.
